# Supplementary material for: Invasive plant species for sustainable security and agricultural biocontrol: integrating allelopathic essential oils and defensive barriers
Source: Front Plant Sci. 2026 Aug 18;17:1666824. doi: 10.3389/fpls.2026.1666824 (PMC13529700; doi:10.3389/fpls.2026.1666824)
Supplement: Supplementary file 1 [file Table1.doc]

**Supplementary Materials**

Detailed Search Strategies for Systematic Review

***Manuscript:*** *"Allelopathic Potential of Essential Oils from Invasive Plant Species in Arid and Semi-Arid Agroecosystems: A Systematic Review and Meta-Analysis for Sustainable Weed Management"*

**Search Period:** January 1, 2010 – December 31, 2024
**Last Search Date:** December 15, 2024
**Total Databases Searched:** 7 + Grey Literature Sources

**S1. Search Strategy Development**

**S1.1 Keyword Development Process**

The search strategy was developed through an iterative process involving:

1. **Preliminary scoping searches** to identify key terminology
2. **Consultation with subject matter experts** in plant ecology and weed science
3. **Analysis of key papers** to extract relevant index terms
4. **Testing and refinement** across multiple databases
5. **Validation** by ensuring retrieval of known relevant studies

**Primary Concept Groups:**

- **Invasive Plants:** invasive plant*, alien plant*, exotic plant*, non-native plant*, weed*, invasive species
- **Essential Oils:** essential oil*, volatile oil*, aromatic oil*, plant extract*, secondary metabolite*
- **Allelopathy:** allelopath*, phytotoxic*, herbicid*, plant growth inhibit*, germination inhibit*
- **Geographic/Climate:** arid, semi-arid, dryland, desert, Mediterranean, subtropical
- **Applications:** weed control, bioherbicide*, natural herbicide*, sustainable agriculture

**S2. Database-Specific Search Strategies**

**S2.1 PubMed/MEDLINE**

**Database URL:** https://pubmed.ncbi.nlm.nih.gov/
**Search Date:** December 15, 2024
**Search Interface:** Advanced Search Builder
**Results Retrieved:** 687 records

**Complete PubMed Search Strategy:**

#1 ("invasive plant*"[Title/Abstract] OR "alien plant*"[Title/Abstract] OR "exotic plant*"[Title/Abstract] OR "non-native plant*"[Title/Abstract] OR "invasive species"[Title/Abstract] OR "invasive weed*"[Title/Abstract])

#2 ("essential oil*"[Title/Abstract] OR "volatile oil*"[Title/Abstract] OR "aromatic oil*"[Title/Abstract] OR "plant extract*"[Title/Abstract] OR "secondary metabolite*"[Title/Abstract] OR "natural product*"[Title/Abstract])

#3 ("allelopath*"[Title/Abstract] OR "phytotoxic*"[Title/Abstract] OR "herbicid*"[Title/Abstract] OR "weed control"[Title/Abstract] OR "plant growth inhibit*"[Title/Abstract] OR "germination inhibit*"[Title/Abstract] OR "bioherbicide*"[Title/Abstract] OR "natural herbicide*"[Title/Abstract])

#4 ("arid"[Title/Abstract] OR "semi-arid"[Title/Abstract] OR "dryland"[Title/Abstract] OR "desert"[Title/Abstract] OR "Mediterranean"[Title/Abstract] OR "subtropical"[Title/Abstract] OR "dry climate"[Title/Abstract])

#5 #1 AND #2 AND #3 AND #4

**Filters Applied:**
- Publication Date: 2010/01/01 to 2024/12/31
- Language: English, French, Spanish
- Article Types: Journal Article, Review, Meta-Analysis
- Species: Plants

**Search Results Breakdown:**
• Initial results: 1,247 records
• After date filter: 892 records
• After language filter: 687 records
• Duplicates removed: 156 records
• Final unique records: 531 records

**S2.2 Scopus**

**Database URL:** https://www.scopus.com/
**Search Date:** December 15, 2024
**Search Interface:** Advanced Search
**Results Retrieved:** 923 records

**Complete Scopus Search Strategy:**

TITLE-ABS-KEY(("invasive plant*" OR "alien plant*" OR "exotic plant*" OR "non-native plant*" OR "invasive species" OR "invasive weed*") AND ("essential oil*" OR "volatile oil*" OR "aromatic oil*" OR "plant extract*" OR "secondary metabolite*" OR "natural product*") AND ("allelopath*" OR "phytotoxic*" OR "herbicid*" OR "weed control" OR "plant growth inhibit*" OR "germination inhibit*" OR "bioherbicide*" OR "natural herbicide*") AND ("arid" OR "semi-arid" OR "dryland" OR "desert" OR "Mediterranean" OR "subtropical" OR "dry climate"))

**Filters Applied:**
- Publication Year: 2010-2024
- Document Type: Article, Review, Conference Paper
- Language: English, French, Spanish
- Subject Area: Agricultural and Biological Sciences, Environmental Science, Biochemistry

**Search Results Breakdown:**
• Initial results: 1,456 records
• After subject area filter: 1,123 records
• After document type filter: 923 records
• Duplicates with PubMed: 287 records
• New unique records: 636 records

**S2.3 Web of Science Core Collection**

**Database URL:** https://www.webofscience.com/
**Search Date:** December 15, 2024
**Search Interface:** Advanced Search
**Results Retrieved:** 756 records

```
Complete Web of Science Search Strategy:

#1 TS=("invasive plant*" OR "alien plant*" OR "exotic plant*" OR "non-native plant*" OR "invasive species" OR "invasive weed*")

#2 TS=("essential oil*" OR "volatile oil*" OR "aromatic oil*" OR "plant extract*" OR "secondary metabolite*" OR "natural product*")

#3 TS=("allelopath*" OR "phytotoxic*" OR "herbicid*" OR "weed control" OR "plant growth inhibit*" OR "germination inhibit*" OR "bioherbicide*" OR "natural herbicide*")

#4 TS=("arid" OR "semi-arid" OR "dryland" OR "desert" OR "Mediterranean" OR "subtropical" OR "dry climate")

#5 #1 AND #2 AND #3 AND #4

Filters Applied:
- Timespan: 2010-2024
- Document Types: Article, Review, Proceedings Paper
- Language: English, French, Spanish
- Web of Science Categories: Plant Sciences, Ecology, Agriculture Multidisciplinary, Environmental Sciences
```

**Search Results Breakdown:**
• Initial results: 1,089 records
• After category filter: 834 records
• After document type filter: 756 records
• Duplicates with previous searches: 298 records
• New unique records: 458 records

**S2.4 CAB Abstracts**

**Database URL:** https://www.cabi.org/publishing-products/cab-abstracts/
**Search Date:** December 15, 2024
**Search Interface:** CAB Direct
**Results Retrieved:** 481 records

**Complete CAB Abstracts Search Strategy:**

(("invasive plant*" OR "alien plant*" OR "exotic plant*" OR "non-native plant*" OR "invasive species" OR "invasive weed*") AND ("essential oil*" OR "volatile oil*" OR "aromatic oil*" OR "plant extract*" OR "secondary metabolite*" OR "natural product*") AND ("allelopath*" OR "phytotoxic*" OR "herbicid*" OR "weed control" OR "plant growth inhibit*" OR "germination inhibit*" OR "bioherbicide*" OR "natural herbicide*") AND ("arid" OR "semi-arid" OR "dryland" OR "desert" OR "Mediterranean" OR "subtropical" OR "dry climate")) IN ti,ab,kw

**Filters Applied:**
- Publication Year: 2010-2024
- Document Type: Journal article, Review article
- Language: English, French, Spanish
- Subject: Crop protection, Plant pathology, Weed science, Plant ecology

**Search Results Breakdown:**
• Initial results: 634 records
• After subject filter: 521 records
• After document type filter: 481 records
• Duplicates with previous searches: 189 records
• New unique records: 292 records

**S2.5 AGRICOLA**

**Database URL:** https://agricola.nal.usda.gov/
**Search Date:** December 15, 2024
**Search Interface:** Advanced Search
**Results Retrieved:** 234 records

**Complete AGRICOLA Search Strategy:**

((invasive plant* OR alien plant* OR exotic plant* OR non-native plant* OR invasive species OR invasive weed*) AND (essential oil* OR volatile oil* OR aromatic oil* OR plant extract* OR secondary metabolite* OR natural product*) AND (allelopath* OR phytotoxic* OR herbicid* OR weed control OR plant growth inhibit* OR germination inhibit* OR bioherbicide* OR natural herbicide*) AND (arid OR semi-arid OR dryland OR desert OR Mediterranean OR subtropical OR dry climate))

**Filters Applied:**
- Publication Date: 2010-2024
- Material Type: Journal Article, Review
- Language: English, French, Spanish
- Subject Terms: Weeds, Plant extracts, Allelopathy, Arid lands

**Search Results Breakdown:**
• Initial results: 298 records
• After material type filter: 234 records
• Duplicates with previous searches: 156 records
• New unique records: 78 records

**S2.6 BIOSIS Previews**

**Database URL:** https://clarivate.com/webofsciencegroup/solutions/biosis-previews/
**Search Date:** December 15, 2024
**Search Interface:** Web of Science Platform
**Results Retrieved:** 189 records

**Complete BIOSIS Previews Search Strategy:**

TS=((invasive plant* OR alien plant* OR exotic plant* OR non-native plant* OR invasive species OR invasive weed*) AND (essential oil* OR volatile oil* OR aromatic oil* OR plant extract* OR secondary metabolite* OR natural product*) AND (allelopath* OR phytotoxic* OR herbicid* OR weed control OR plant growth inhibit* OR germination inhibit* OR bioherbicide* OR natural herbicide*) AND (arid OR semi-arid OR dryland OR desert OR Mediterranean OR subtropical OR dry climate))

**Filters Applied:**
- Timespan: 2010-2024
- Document Types: Article, Review
- Language: English, French, Spanish
- Research Areas: Plant Sciences, Environmental Sciences & Ecology, Agriculture

**Search Results Breakdown:**
• Initial results: 267 records
• After research area filter: 189 records
• Duplicates with previous searches: 134 records
• New unique records: 55 records

**S2.7 Google Scholar**

**Database URL:** https://scholar.google.com/
**Search Date:** December 15, 2024
**Search Interface:** Advanced Search
**Results Retrieved:** 200 records (first 200 results per search string)

**Google Scholar Search Strings:**

**Search String 1:**
"invasive plant" "essential oil" allelopathy arid weed control
Results: 200 (first 200 examined)

**Search String 2:**
"alien plant" "volatile oil" phytotoxic semi-arid bioherbicide
Results: 200 (first 200 examined)

**Search String 3:**
"exotic plant" "plant extract" "germination inhibition" Mediterranean
Results: 200 (first 200 examined)

**Filters Applied:**
- Publication Date: 2010-2024
- Language: English, French, Spanish
- Document Type: Articles only (excluding patents and citations)

**Search Results Breakdown:**
• Total results examined: 600 records
• Relevant records identified: 89 records
• Duplicates with database searches: 67 records
• New unique records: 22 records

**S3. Grey Literature and Additional Sources**

**S3.1 Thesis and Dissertation Repositories**

**ProQuest Dissertations & Theses Global**

**Search Strategy:**
noft(invasive plant* OR alien plant* OR exotic plant*) AND noft(essential oil* OR volatile oil*) AND noft(allelopath* OR phytotoxic* OR weed control) AND noft(arid OR semi-arid OR Mediterranean)

**Results:** 23 dissertations identified, 8 relevant after screening

**S3.2 Conference Proceedings**

**Key Conferences Searched:**

- International Weed Science Congress (2010-2024)
- International Allelopathy Society Congress (2010-2024)
- European Weed Research Society Symposium (2010-2024)
- International Conference on Invasive Species (2010-2024)
- Mediterranean Plant Protection Organization Conferences (2010-2024)

**Results:** 34 conference abstracts identified, 12 with sufficient data for inclusion consideration

**S3.3 Government and Institutional Reports**

**Sources Searched:**

- USDA Agricultural Research Service Reports
- European Environment Agency Publications
- FAO Technical Reports on Invasive Species
- CABI Invasive Species Compendium
- National Agricultural Research Organization Reports

**Results:** 15 technical reports identified, 3 with relevant quantitative data

**S4. Citation Searching**

**S4.1 Backward Citation Searching**

Reference lists of all included studies and relevant review articles were manually screened for additional eligible studies.

**Backward Citation Results:**
• Studies screened: 47 included studies + 23 relevant reviews
• Additional references identified: 156 records
• New eligible studies found: 8 studies
• Most productive sources: Allelopathy Journal, Weed Science, Industrial Crops and Products

**S4.2 Forward Citation Searching**

Forward citation searching was conducted using Google Scholar and Web of Science for key studies in the field.

**Forward Citation Results:**
• Key studies tracked: 15 seminal papers (2010-2020)
• Citing articles screened: 234 records
• New eligible studies found: 5 studies
• Search completed: December 2024

**S5. Search Results Summary**

| **Database/Source** | **Search Date** | **Records Retrieved** | **After Deduplication** | **Unique Contribution** | **Final Included** |
| --- | --- | --- | --- | --- | --- |
| PubMed/MEDLINE | Dec 15, 2024 | 687 | 531 | 531 | 18 |
| Scopus | Dec 15, 2024 | 923 | 636 | 636 | 15 |
| Web of Science | Dec 15, 2024 | 756 | 458 | 458 | 8 |
| CAB Abstracts | Dec 15, 2024 | 481 | 292 | 292 | 4 |
| AGRICOLA | Dec 15, 2024 | 234 | 78 | 78 | 1 |
| BIOSIS Previews | Dec 15, 2024 | 189 | 55 | 55 | 1 |
| Google Scholar | Dec 15, 2024 | 600* | 22 | 22 | 0 |
| **Database Subtotal** | - | **3,870** | **2,072** | **2,072** | **47** |
| Grey Literature | Dec 10-15, 2024 | 72 | 23 | 23 | 0 |
| Citation Searching | Dec 16-18, 2024 | 390 | 13 | 13 | 0 |
| **TOTAL** | **-** | **4,332** | **2,108** | **2,108** | **47** |

**Google Scholar: First 200 results examined for each of 3 search strings
Final screening: 2,108 unique records → 312 full-text assessed → 47 included*

**S6. Search Strategy Validation**

**S6.1 Known Item Searching**

To validate the comprehensiveness of our search strategy, we tested whether it could retrieve a set of known relevant studies identified through preliminary scoping:

**Validation Results:**
• Known relevant studies tested: 15 key papers
• Successfully retrieved: 14/15 (93.3%)
• Missing study reason: Published in non-indexed regional journal
• Additional search conducted: Manual search of regional journals

**S6.2 Search Strategy Peer Review**

The search strategy was independently reviewed by:

- Information specialist with expertise in agricultural databases
- Subject matter expert in plant allelopathy
- Systematic review methodologist

**Peer Review Feedback Incorporated:**
• Added "natural product*" to essential oil terms
• Included "dry climate" as geographic descriptor
• Expanded to include "bioherbicide*" terminology
• Added BIOSIS Previews database for comprehensive coverage

**S7. Search Update Strategy**

**S7.1 Search Alert Setup**

To monitor for new relevant publications during the review process, search alerts were established in:

- PubMed (saved search with email alerts)
- Scopus (search alert with monthly updates)
- Web of Science (saved search with alerts)
- Google Scholar (citation alerts for key papers)

**S7.2 Pre-Publication Update**

A final search update will be conducted immediately before manuscript submission to capture any newly published relevant studies.

**Update Search Protocol:**
• Timing: Within 30 days of submission
• Databases: All primary databases (PubMed, Scopus, Web of Science, CAB Abstracts)
• Date range: From last search date to update date
• Screening: Same criteria as original search
• Integration: New studies incorporated if they meet inclusion criteria

**S8. Search Limitations and Considerations**

**S8.1 Language Limitations**

While our search included English, French, and Spanish terms, relevant studies published in other languages (particularly Arabic, Portuguese, and Chinese) may have been missed. However, major international databases predominantly index English-language publications, and key findings are typically published in internationally accessible journals.

**S8.2 Publication Bias Considerations**

Our search strategy was designed to minimize publication bias by:

- Including grey literature sources
- Searching conference proceedings
- Examining thesis repositories
- Using broad search terms to capture negative results
- Conducting comprehensive citation searching

**S8.3 Database Coverage Limitations**

Some regional databases and specialized journals may not be fully covered by our selected databases. However, the combination of seven major databases plus grey literature searching provides comprehensive coverage of the relevant literature.

**S9. Reproducibility Information**

**Search Documentation Package Includes:** • Complete search strategies for all databases • Search result export files with metadata • Deduplication process documentation • Screening decision logs • Full-text retrieval records • Data extraction forms • Quality assessment tools **Available Upon Request:** • Raw search result files (.ris, .bib formats) • Detailed screening logs with reasons for exclusion • Inter-rater reliability statistics • Search strategy development notes • Database interface screenshots

***Contact Information:*** *All search strategies, results files, and documentation are available from the corresponding author upon reasonable request to support reproducibility and transparency of this systematic review.*

**Supplementary Materials - Search Strategies**
Systematic Review: Allelopathic Potential of Essential Oils from Invasive Plant Species
Last Updated: December 2024 | Total Pages: 12 | Total Databases: 7 + Grey Literature
